# The centrality of temperament to the research domain criteria (RDoC): The earliest building blocks of psychopathology

**DOI:** 10.1017/S0954579421000511

**Published:** 2021-12

**Authors:** Brendan Ostlund, Sarah Myruski, Kristin Buss, Koraly E. Pérez-Edgar

**Affiliations:** 1 Department of Psychology, The Pennsylvania State University, University Park, US; 2 Department of Human Development and Family Studies, The Pennsylvania State University, University Park, US

**Keywords:** developmental psychopathology, early childhood, infancy, research domain criteria (RDoC), temperament

## Abstract

The research domain criteria (RDoC) is an innovative approach designed to explore dimensions of human behavior. The aim of this approach is to move beyond the limits of psychiatric categories in the hope of aligning the identification of psychological health and dysfunction with clinical neuroscience. Despite its contributions to adult psychopathology research, RDoC undervalues ontogenetic development, which circumscribes our understanding of the etiologies, trajectories, and maintaining mechanisms of psychopathology risk. In this paper, we argue that integrating temperament research into the RDoC framework will advance our understanding of the mechanistic origins of psychopathology beginning in infancy. In illustrating this approach, we propose the incorporation of core principles of temperament theories into a new “life span considerations” subsection as one option for infusing development into the RDoC matrix. In doing so, researchers and clinicians may ultimately have the tools necessary to support emotional development and reduce a young child’s likelihood of psychological dysfunction beginning in the first years of life.

Initiated over a decade ago (Insel et al., 2010), the research domain criteria (RDoC) framework was introduced in response to a growing disconnect between clinical neuroscience and the classification used to understand psychiatric disorder. In particular, the approach taken by clinical neuroscience focused on dynamic and elastic processes that functioned over time to generate and sustain patterns of cognition, emotion, and behavior. The data generated by this approach had limited translation to front-line treatment as they were generally incompatible with a traditional nosological approach that uses diagnostic categories determined by a combinatorial selection of associated symptoms. RDoC aims to provide researchers with a data-driven, theoretically agnostic framework to integrate behavior and biology across levels of analysis. This approach reframed research priorities with the goal of yielding more nuanced classification of psychological health and dysfunction, emphasizing mechanistic validity rather than diagnostic reliability, a core feature of the prevailing approach. RDoC is currently considered a guide for basic research, although long-term applications aim to inform individualized treatment (Cuthbert & Insel, 2010). Despite its promise, RDoC has core gaps in approach that may limit its utility for prevention and intervention. Specifically, the RDoC matrix undervalues ontogenetic development, which circumscribes our understanding of the etiologies, trajectories, and maintaining mechanisms of psychopathology risk.

In this paper, we provide evidence to support the claim that integrating temperament research – defined as a young child’s proclivities in experiencing and demonstrating affect, attention, activity, and regulation (Shiner et al., 2012) – into the RDoC framework will advance our understanding of the mechanistic origins that underlie psychopathology from early childhood and shape trajectories well into adulthood. Whether defined by fine-grained emotions (e.g., anger) or broad phenotypes (e.g., dysregulated fear), a young child’s temperament can serve as an indicator of potential psychological health or dysfunction that is evident as early as the first year of life. Decades of research in this area has aimed to describe the genetic, neural, cognitive, emotional, and behavioral manifestations of temperament traits, while considering their interaction over time in response to varied early experiences. Indeed, temperament traits reliably predict childhood psychopathology risk, often as a function of internal (cognitive, emotional) or external (environmental) influences (see Stifter & Dollar, 2016 for review). Adopting a trait-based approach to early individual differences in psychopathology risk will provide a practical guide for researchers to integrate a life span neurodevelopmental perspective into the largely static RDoC matrix. A better understanding of the etiology and early emergence of psychopathology will, in turn, inform practice with both children and adults as clinicians incorporate first-line treatments sensitive to specific clinical trajectories.

The ideas we present here build upon recent calls for integrating developmental psychopathology and RDoC perspectives via a trait-based, “neural system first” approach to pediatric research (Beauchaine & Hinshaw, 2020; Garber & Bradshaw, 2020). Temperament traits reflect transdiagnostic, neurobiologically based vulnerabilities that transact with environmental influences to shape behavioral development (e.g., Beauchaine, Zisner, & Sauder, 2017; Clauss, Avery, & Blackford, 2015). This transaction between the individual and the environment is a central tenet of life span approaches to psychopathology risk (Crowell, Puzia, & Yaptangco, 2015). In illustrating this approach, we focus on birth through preschool age due to the rapid, widespread, and environmentally sensitive neural and behavioral development that defines this period of life. Indeed, the dynamic biology–environment interplay that takes place during this period yields individual differences in neurobehavior that support emotion, cognition, and behavior for years to follow. Across early life, these differences are thought to be elaborated upon and biologically embedded via epigenetic, morphological, and physiological mechanisms (Gottlieb, 2007). The protracted rate of neurodevelopment (beginning in utero) leaves a young child’s developing brain sensitive to proximal and distal external factors, such as positive and negative aspects of the caregiving context (e.g., Bick & Nelson, 2017; Cui et al., 2018). Leveraging detailed information on specific risk traits during these early years of vulnerable brain development may aid RDoC-focused researchers and clinicians in identifying transdiagnostic and modifiable targets for intervention that reduce a young child’s psychopathology risk prior to symptom onset (Wakschlag et al., 2018).

## The RDoC Framework

Despite its relatively short tenure, the composition, merits, and shortcomings of the RDoC framework have been discussed extensively (Beauchaine & Hinshaw, 2020; Casey, Oliveri, & Insel, 2014; Cuthbert & Insel, 2010; Franklin, Jamieson, Glenn, & Nock, 2015; Garber & Bradshaw, 2020; Insel et al., 2010; Lilienfeld, 2014). A comprehensive review of the RDoC literature is beyond the scope of this paper. We briefly describe the RDoC framework and highlight major critiques as they pertain to research with pediatric samples.

The core component of the RDoC approach is the matrix, which provides researchers with a structured guide for investigations into basic dimensions of human functioning. The RDoC matrix consists of six major domains – negative valence, positive valence, cognitive, social processes, arousal/regulatory, and sensorimotor systems – that are further divided into several more specific constructs (e.g., acute threat, reward valuation, affiliation, and attachment), each of which is operationalized across multiple levels of analysis – molecules, cells, neural circuits, physiology, behavior, and self-report. The matrix is directly informed by research and therefore designed to be continuously evolving as new findings emerge (Cuthbert & Insel, 2010).

RDoC’s emphasis on dimensional constructs permits researchers to explore patterns of functioning ranging from normal to atypical both within and between constructs. RDoC also promotes the identification of biobehavioral vulnerabilities that cut across diagnostic categories, acknowledging the heterogeneity inherent to psychological dysfunction. For example, heightened response to threat, disrupted reward processing, and attentional biases have each been implicated as mechanisms that contribute to both anxiety and mood disorders (e.g., Buss & Qu, 2018; Casey et al., 2014; Dillon et al., 2014; Jarcho & Guyer, 2018). These areas of impairment cut across dimensions of positive valence, negative valence, and cognitive systems and are controlled by cortical–subcortical neural circuitry, which, in turn, is thought to modulate peripheral physiology and behavior. The RDoC domains (rows) and levels of analysis (columns) foster the identification of mechanisms underlying psychological health and dysfunction. However, since the triggers and consequences of maladaptive traits vary widely across individuals, these mechanisms must also be contextualized within the environment and across development in order to capture fully the impact on individual functioning.

The RDoC matrix has yielded promising insight into adult psychopathology; nevertheless, several limitations are worth noting. First, certain units of analysis are inextricably linked (e.g., circuits and physiology), raising questions as to whether these systems should be defined independently in the framework. Second, impairments across domains of functioning (e.g., negative valence systems, cognitive systems) exhibit strong convergent validity but often lack discriminant validity. Deficits in these systems frequently co-occur and are shared across psychopathologies, which serves the transdiagnostic goals of the framework. However, some (e.g., Shankman & Gorka, 2015) argue that more focus is needed on how impairments across systems diverge as predictors of symptom clusters. For example, threat–safety discrimination, a component of fear conditioning relevant to the negative valence system, has been associated with familial risk for anxiety but not depression, while reduced sensitivity to reward, part of the positive valence system, was linked to depression but not anxiety (Nelson et al., 2013). More research clarifying the specificity of deficits within and across domains as predictors of psychological dysfunction is warranted.

Another challenge comes from the practicalities of conceptualizing and conducting RDoC-informed research without reverting to the prevailing model of clinical science. Indeed, despite being the target of investigation, biological and behavioral dysfunctions are still frequently analyzed as correlates of symptom profiles from traditional diagnostic categories, often controlling for comorbidities, eschewing the very complexity that the National Institute of Mental Health (NIMH) aims to capture. Despite explicit statements that RDoC is not intended as a diagnostic tool, attempts to link biobehavioral vulnerabilities with specific disorders in a one-to-one manner are common. Third and finally, the strong emphasis on biological underpinnings in the RDoC framework has been interpreted by some as biological reductionism (see for example, Berenbaum, 2013), framing expectations that specific units of analysis, particularly neural circuitry, should be prioritized when examining mechanisms underpinning psychological dysfunction.

In presentation and conceptualization, the RDoC matrix is also to be linked to developmental and environmental considerations. This link, however, has garnered far less attention from researchers. As the RDoC documentation states, “many areas of the child psychopathology literature (such as reward sensitivity, emotional dysregulation, behavioral inhibition) serve as a more compatible model for a dimensionally based approach compared to the highly specified categories of adult psychopathology” (NIMH, 2018). To this end, there has been a recent push to incorporate developmental perspectives more explicitly in the RDoC approach, particularly with regard to developmental pathophysiology (Franklin et al., 2015; Mittal & Wakschlag, 2017). Still, the RDoC matrix presents dimensions of human behavior as largely static, lacking clear guidelines on how developmental considerations should be integrated into the matrix.

RDoC’s atheoretical approach was intended to free researchers from constraints of prevailing clinical diagnostic categories. For example, rather than focusing on correlates of depressive disorder, researcher A may choose to investigate the neurobiological underpinnings of ruminative thoughts, while researcher B explores trajectories of pathophysiology related to disrupted sleep, both of which are symptoms of depression. Advancements from each of these hypothetical researchers would provide insight into processes central to many forms of psychological dysfunction beyond simply depression, and may ultimately identify malleable points of intervention.

By situating itself as an atheoretical approach, however, RDoC may have limited its capacity to advance knowledge on the ontogeny of psychological dysfunction. Clearly articulating one’s theoretical perspective serves two purposes: to consolidate extant evidence and to propose plausible explanations that can be subsequently tested. RDoC need not exclusively adhere to a single theoretical perspective. Flexible integration of principles from multiple theoretical perspectives may provide a robust representation of a construct of interest, facilitating multidisciplinary research that furthers knowledge on a topic. Integrating complementary theoretical perspectives is particularly important when considering developmental processes, given the complexity inherent to understanding social, emotional, cognitive, and biological system maturation across the life span. Thus, to better understand development and psychopathology risk, RDoC may benefit from a move away from its atheoretical approach and instead consider how multiple, neurodevelopmentally informed theories can be incorporated into the framework.

These limitations may contribute to the protracted integration of development into the RDoC matrix. To harness the advantages RDoC provides for examining origins and mechanisms of psychological functioning, researchers might consider trait vulnerabilities that are predictive of, but not restricted to, adult psychopathology presentation. A trait-based, “neural systems first” approach to childhood psychopathology risk (Beauchaine & Constantino, 2017; Beauchaine & Hinshaw, 2020) leverages detailed, multiple-levels-of-analysis information on a specific trait (e.g., anger) and considers it in dynamic interaction with other well-described traits (e.g., cognitive control) over time. This approach emphasizes the utility of neurobiologically informed, transdiagnostic traits as starting points to understand childhood psychopathology risk. In this way, temperament researchers are uniquely positioned to infuse development into the RDoC matrix – emphasizing individual differences in dimensional neurobehavioral proclivities examined with an appreciation for maturational and environmental influences that shape functioning over time and across levels of analysis.

## Temperament in Early Life

Temperament traits across early childhood provide the foundation for many facets of adult functioning – from cognition, motivation, and attention to personality and psychopathology (Pérez-Edgar & Fox, 2018; Rothbart & Bates, 2006; Stifter & Dollar, 2016). Contemporary definitions of temperament highlight biologically based individual differences in behavior and emotionality that are reflected in moderately stable traits. The emphasis is on “moderately,” as temperament traits are malleable and reflect maturational and environmental influences across early life. Thus, trait expression reflects a complex interplay between genetic disposition, neurobehavioral activity, and the early environment, with an emphasis on the caregiving context as a determinant of functional development (Shiner et al., 2012).

Modern temperament research has been informed by two core principles (Shiner et al., 2012; Stifter & Dollar, 2016), described specifically below.

### (a) Modern temperament research focuses on the individual through development.

A central aim of temperament research is to describe traits and phenotypes that predict psychological health and dysfunction. In doing so, this work strives to characterize a child’s strengths and weaknesses in light of environmental demands within and across developmental windows. This information, in turn, helps to predict developmental trajectories for specific processes and outcomes. Indeed, it is this predictive and explanatory power that makes temperament research particularly beneficial within the context of RDoC. Inherent in temperament research is also an acknowledgement that manifest emotions and behaviors may change over time in conjunction with trait or skill development in other domains. Supporting the current paper’s focus on early development, a rich literature base demonstrates that temperament in the first 5 years of life is characterized by both stability and change (e.g., Braungart-Rieker, Hill-Soderlund, & Karrass, 2010; Bridgett, Laake, Gartstein, & Dorn, 2013; Gartstein & Hancock, 2019), which provides the necessary malleability for scientific and clinical utility.

### (b) Modern temperament research cuts across multiple levels of analysis.

Temperament research is by its very nature an investigation into individual difference across interdependent levels of analysis. Historically, the temperament literature implemented an RDoC approach, even before there was an RDoC. That is, while the literature is rooted in extensive descriptions of behavior, temperament traits have been linked to interrelated structural and functional differences across domains in the developing brain. Indeed, a core tenet of temperament research is that traits are biologically based and, to varying degrees, heritable (Saudino & Micalizzi, 2015; Shiner et al., 2012). Converging evidence from epidemiology and developmental psychobiology supports both genetic and experience-dependent (possibly beginning in utero) pathways to the development of temperament traits via adjustments to the structure and function of early neurobehavioral systems. Many temperament approaches were rooted in models that linked biological functioning and behavioral profiles – as can be seen in the amygdala model of behavioral inhibition (Kagan, Reznick, & Snidman, 1987; discussed below). This approach is embedded in the temperament literature as seen in systematic studies of sympathetic, parasympathetic, and neuroendocrine activity at rest and in response to challenge (see e.g., Buss, Morales, Cho, & Philbrook, 2015; Miskovic & Schmidt, 2012; Nigg, 2006; Stifter & Dollar, 2016 for reviews). Biological and behavioral activity does not, however, occur in a vacuum. To this end, contemporary temperament research also aims to understand the way in which environmental factors, such as parenting or poverty, modulate the trajectory of trait expression (and possible psychopathology risk) for a young child.

Inherent to each of the principles is the notion that temperament researchers seek to understand the processes through which traits manifest and change over time, whether it is due to maturational or environmental influences, or an interaction (or transaction) between the two. These core principles are echoed across the varying theoretical perspectives on temperament, which can broadly be categorized into deductive (theory informs interpretation) and inductive (data informs theory) approaches (see Fu & Pérez-Edgar, 2015 for review).

Perhaps no deductive approach to temperament aligns with these guiding principles, or the structure of the RDoC matrix, as directly as Rothbart’s psychobiological model (Rothbart & Derryberry, 1981). A dimensional approach, Rothbart’s model argues that reactivity and self-regulation are neurobiological processes that are intimately linked and can be operationalized via three broad temperament dimensions, namely, negative affect, surgency, and effortful control. These broad dimensions can be divided into finer grained traits (e.g., anger/frustration, activity level, inhibitory control) that develop over time. In particular, this perspective emphasizes both micro-longitudinal –threshold, latency, and rate of recovery of a behavioral response – and macro-longitudinal – maturational and environmental influences on trait expression (e.g., Braungart-Rieker et al., 2010; Gartstein & Hancock, 2019) – change processes in its conceptualization of temperament.

The integration of temporal dynamics is reflected in the model of temperament by Goldsmith and Campos as well (Goldsmith & Campos, 1982), which focused on developing emotional capacities as a defining feature of temperament. This perspective posits that emotion and the regulation of emotion are intertwined concepts that are difficult to disentangle without consideration of the temporal dynamics of emotions (Campos, Frankel, & Camras, 2004; Cole, Martin, & Dennis, 2004; see Pérez-Edgar, 2019 for counter argument). These authors have also argued for the heritable nature of emotional expression in early childhood, a view that is integral to Buss and Plomin’s criterion model of temperament as well (Buss & Plomin, 1975; 1984). Each of these models converges with the suggestion that trait expression is, to varying degree, inherited and appears as a stable profile in infancy, even though they may differ in what constitutes temperament traits in the first years of life.

These dimensional models of temperament are contrasted by typological models. Typological models consider a behavioral phenotype as an emergent property that is more than the sum of its constituent behaviors, and this leads to categories, or “types,” of children. This approach is exemplified by the behavioral-style model of Thomas and Chess (Thomas & Chess, 1977) and Kagan’s biotypological model (Kagan, 1994), both of which may be considered inductive approaches. In their foundational temperament study, Chess and Thomas (Chess & Thomas, 1984; Thomas & Chess, 1977) categorized (most) children into one of three categories – *easy*, *slow to warm*, and *difficult* – based on nine dimensions of behavior identified during extensive interviews with parents. With these types in mind, Thomas and Chess argued that the most adaptive childhood outcomes would occur when the temperamental qualities of a child were congruent with environmental characteristics suited for that temperament type, a concept they referred to as “goodness of fit.” Maladaptive childhood outcomes were thought to be a consequence of a mismatch between a young child’s proclivities and their environment, rather than either of these factors in isolation (Thomas & Chess, 1977).

Kagan and colleagues (Kagan, 2018; Pérez-Edgar & Fox, 2018) took a different approach to identifying another behavioral phenotype, behavioral inhibition. Based on initial observations of toddlers, and emerging animal research, Kagan and colleagues proposed that individual differences in this behavioral phenotype were attributable to a hyper-reactive amygdala to novelty (Kagan et al., 1987). Over the past four decades, researchers have continued to garner data to characterize behavioral inhibition, a temperament trait defined by reticence in response to unexpected or unfamiliar stimuli. This trait is identifiable by the age of 2 (Garcia-Coll, Kagan, & Reznick, 1984), with a developmental precursor, high negative reactivity, evident in the first year of life among approximately 20% of infants (Fox, Henderson, Rubin, Calkins, & Schmidt, 2001; Fox, Snidman, Haas, Degnan, & Kagan, 2015). A continued emphasis on a multiple-levels-of-analysis approach has led to detailed information on the neural, physiological, cognitive, emotional, and social correlates and underpinnings of this temperament trait (see Pérez-Edgar & Fox, 2018 for a detailed discussion). This work is particularly useful from the RDoC perspective as behavioral inhibition is one of the most reliable predictors of childhood internalizing of symptoms, specifically increasing the risk for social anxiety six-fold (Clauss & Blackford, 2012).

Building on this line of research, Buss and colleagues (Buss, 2011; Buss et al., 2013; Buss, Davis, Ram, & Coccia, 2018) provided support for a dysregulated fear profile – a pattern of behavior characterized by high levels of fear in both low- and high-threat contexts. This construct expands upon Kagan’s behavioral inhibition profile by identifying high-risk toddlers based on when (e.g., low-threat situation) and what type of fearful behavior is displayed, in addition to the intensity of a fear response (Buss & Kiel, 2013). In this way, dysregulated fear considers situational specificity as a key component to identifying young children at risk for psychopathology. In doing so, researchers have shown that dysregulated fear is a strong predictor of childhood internalizing of symptoms above and beyond behavioral inhibition (Buss, 2011; Buss et al., 2013; 2018). It is worth noting that the constructs of behavioral inhibition and dysregulated fear have each been identified via dimensional analyses and latent profile analysis (Buss, 2011; Loken, 2004), lending support for their robust representation as predictors of psychopathology risk in early life.

More recently, researchers have sought to incorporate advanced statistical methods to identify a finite number of distinct, homogeneous groups of children defined by a shared constellation of behaviors (e.g., Beekman et al., 2015; Karalunas et al., 2014; Ostlund et al., in press; Scott et al., 2016). In many ways, this data-driven approach leverages the strengths of both continuous and categorical conceptualization of temperament. Namely, continuous ratings of several temperament traits (typically parent-report data, but also observed data) are used to identify a finite number of subgroups of children (typically 3–5) who have constellations of traits in common, via person-centered analytic approaches, such as latent profile analysis. A non-exhaustive list of sample characteristics for studies that have used this approach with children under the age of 5 is presented in the Supplement.

Studies using this approach broadly categorize young children into six temperament profiles. Akin to the behavioral inhibition typology identified by Kagan and colleagues, one commonly identified profile is defined by high fearfulness and low regulatory abilities (Beekman et al., 2015; Caspi & Silva, 1995; Gartstein et al., 2017; Janson & Mathiesen, 2008; Putnam & Stifter, 2005; Sanson et al., 2009; Usai, Garello, & Viterbori, 2009; Van Den Akker, Deković, Prinzie, & Asscher, 2010), and may thus be considered an *inhibited/fearful* group of young children. Another commonly observed profile, which might be referred to as *negative reactive*, is similar to the *inhibited/fearful* profile with the exception that, rather than being exclusively defined by extreme fear, these children exhibit high levels of negative affect (e.g., anger, sadness) more broadly across contexts (Beekman et al., 2015; Janson & Mathiesen, 2008; Komsi et al., 2006; Lin, Ostlund, Conradt, & Lagasse, 2018; Ostlund et al., in press; Planalp & Goldsmith, 2020; Prokasky et al., 2017). Researchers have also consistently observed what might be considered a *dysregulated/irritable* profile, that characterizes young children who display high negative affect, particularly anger, paired with high activity levels (Beekman et al., 2015; Gartstein et al., 2017; Lin et al., 2018; Ostlund et al., in press; Prokasky et al., 2017; Sanson et al., 2009), reminiscent of trait irritability (Beauchaine et al., 2017; Wakschlag et al., 2018).

Conversely, an *exuberant/fearless* profile characterized by high levels of positive affect, activity, and approach oriented behavior has also been observed (Gartstein et al., 2017; Prokasky et al., 2017; Putnam & Stifter, 2005; Sanson et al., 2009; Usai et al., 2009; Van Den Akker et al., 2010). Lastly, many studies using this approach observe either an *average/low reactive* profile, defined by a modest level of emotionality, attention, and activity (Beekman et al., 2015; Caspi & Silva, 1995; Gartstein et al., 2017; Janson & Mathiesen, 2008; Lin et al., 2018; Planalp & Goldsmith, 2020; Prokasky et al., 2017; Putnam & Stifter, 2005; Usai et al., 2009; Van Den Akker et al., 2010), or a *well-regulated* profile, defined by relatively low levels of negative affect as well as high levels of regulatory abilities, such as effortful control (Beekman et al., 2015; Caspi & Silva, 1995; Gartstein et al., 2017; Janson & Mathiesen, 2008; Komsi et al., 2006; Lin et al., 2018; Planalp & Goldsmith, 2020; Prokasky et al., 2017).

The replicability of these temperament profiles despite differences in methodology (e.g., laboratory assessment, parent-report questionnaire), age, and risk status (e.g., prenatal substance exposure, Lin et al., 2018) underscores their robustness in early childhood.

## Integrating Temperament, Development, and RDoC

We believe temperament research can advance both the refinement and application of RDoC as a conceptual structure and research tool in two meaningful ways: namely, through (a) the integration of principles of temperament theories (i.e., focus on individual over time and across levels of analysis) to inform etiologies, mechanisms, and trajectories of RDoC constructs in early childhood and (b) the identification of transdiagnostic risk traits and phenotypes that are evident prior to preschool age that reliably predict childhood psychopathology risk.

### Illustrative examples

As stated by other developmental psychopathologists (Beauchaine & Hinshaw, 2020; Garber & Bradshaw, 2020), the RDoC matrix would benefit from changes that embrace the complexity inherent in developmental research. One possibility would be to add a developmental qualifier that acknowledges temperamental precursors and trajectories that may contribute to individual differences to each RDoC construct. It may also be useful for identifying what systems are involved and/or might interact to shape the development of that temperamental precursor. For example, a qualifier could be added to describe temperament traits that are relevant to a specific RDoC construct, as well as when they emerge, how they change over time, and what role they play as interrelated neurodevelopmental antecedents to the construct. This approach would highlight gaps in our understanding of a developmental precursor to an RDoC construct, serving as a guide for future research endeavors.

These developmental qualifiers may also help distinguish behaviors that might be considered developmentally appropriate (e.g., the occasional temper tantrum among 2-year-olds) from worrisome patterns (e.g., prolonged tantrums at age 5), as well as points in development when behaviors move from normative to potentially problematic. Similarly to the RDoC matrix at large, these developmental qualifiers would evolve as additional longitudinal research is conducted and consensus about the causes, correlates, and contributions of specific traits is reached. Indeed, this approach would facilitate a seamless, semi-realtime integration of results from large-scale National Institutes of Health studies, such as the Environmental Influences on Child Health Outcomes, Adolescent Brain Cognitive Development, or Healthy Brain and Child Development projects, into the RDoC matrix. Crucially, no study to date has prospectively examined whether the inclusion of early emerging temperament traits into the RDoC framework will advance our understanding of childhood psychopathology risk. This reflects a gap in the literature and an essential next step for future research.

Let us consider two RDoC constructs from the negative valence system, frustrative nonreward and acute threat, as examples to illustrate how temperament research, beginning in the first year of life, can be incorporated as a developmental qualifier on the RDoC matrix. The frustrative nonreward and acute threat constructs are conceptually similar to irritability and behavioral inhibition, respectively. These two temperament traits are commonly included in ontogenetic models of developmental psychopathology (e.g., Beauchaine, 2015; Crowell et al., 2015). A comprehensive review of the neural and physiological mechanisms that contribute to childhood irritability and behavioral inhibition is beyond the scope of this paper. Thus, we note a few key findings from the literature to highlight the utility of core principles described above to infuse development into the RDoC matrix.

### Irritability: A trait antecedent of frustrative nonreward

**Figure 1.**
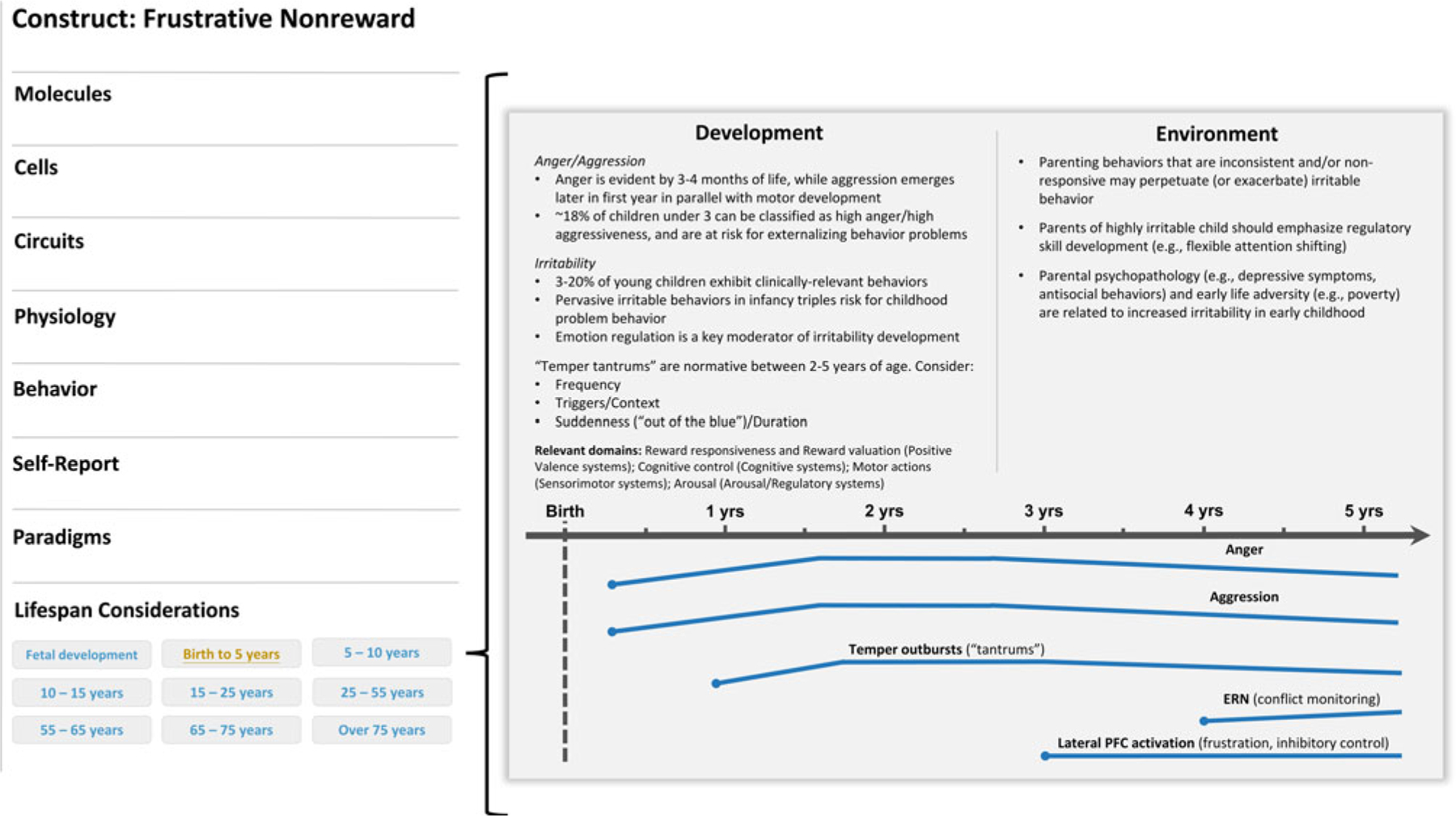
Illustrative example of a developmental qualifier for the frustrative nonreward (negative valence system) construct. A development subsection (“life span considerations”) could be added below the currently listed levels of analysis on the construct page (left panel). In this example, a researcher clicked on the “birth to 5 years” tab and was directed to a new screen that describes developmental considerations available for this construct in this age range. In this case, aspects of trait irritability would be described and, when applicable, visually represented across time. The point in development when a construct could be reliably measured would be indicated on the timeline with a dot. Moreover, various colors could be incorporated to indicate normative and high-risk profiles or trajectories. Relevant cross-domain considerations would also be noted.

Frustrative nonreward is defined by negative reactivity in response to repeated failures at reward attainment (NIMH, 2018). Viewed through a developmental lens, this construct comprises emotional (anger, irritability) and behavioral (reactivity, aggression) expressions that may manifest differently based on a child’s age (Figure 1).

Irritability is a dimensional phenotype conceptualized as the propensity to react to a blocked goal with anger and agitation. In early childhood, irritability is often accompanied by elevated aggression or temper outbursts, which can be maladaptive if inconsistent with context (i.e., “out of the blue”) or persistent (e.g., Denham et al., 2012). The roots of both anger and aggression can be traced to infancy. Anger is evident in the first months of life, while aggression tends to emerge later in infancy in parallel with motor development; both constructs are thought to peak in toddlerhood and decrease thereafter in conjunction with the development of self-regulatory skills (Braungart-Rieker et al., 2010; Gartstein & Hancock, 2019; Hay et al., 2014; Lorber, Del Vecchio, & Slep, 2015). Importantly, the type, frequency, and intensity of the behaviors tend to manifest differently over the first 5 years of life, both within and between individuals (e.g., Denham, Lehman, & Moser, 1995; Eisenberg et al., 1997; Liu et al., 2018). Capturing these variations may help to identify patterns associated with later psychopathology (e.g., Damme, Wakschlag, Briggs-Gowan, Norton, & Mittal, 2021). Using a person-centered approach, Perra, Paine, and Hay (2020) found that, from 6 to 36 months of age, three subgroups of infants could be identified based on their levels of anger/aggressiveness. One group (18% of the sample) showed exceptionally high levels of anger and physical force at each time point, and were at elevated risk for externalizing psychopathology at age 7.

High levels of irritability in infancy have been shown to predict behavior problems in childhood (Winsper & Wolke, 2014), while childhood irritability has been linked to more functional impairment and internalizing symptoms later in development (e.g., Copeland, Angold, Costello, & Egger, 2013; Dougherty et al., 2013a, 2013b, 2015; Stringaris & Goodman, 2009). Irritability tends to peak by preschool age, then decreases and remains stable in later childhood and through adulthood (e.g., Caprara, Paciello, Gerbino, & Cugini, 2007; Wakschlag et al., 2012). Importantly, high levels of irritability in early life underpin multiple psychological disorders, including disruptive mood dysregulation disorder, oppositional defiant disorder, and attention deficit/hyperactivity disorder (Beauchaine et al., 2017). Recent findings from Damme and colleagues (Damme et al., 2021) suggest that the course of irritability over early development may also influence manifest psychopathology in late childhood. In other words, children with high and stable levels of irritability in preschool (*M*_age_ = 4.17 years) and elementary school (*M*_age_ = 6.95 years) were reported to show more externalizing behavior in pre-adolescence. In contrast, children with high but decreasing levels of irritability across these time points displayed more internalizing behavior in pre-adolescence (Damme et al., 2021). Together, these findings suggest that clinical prediction may be improved when the developmental course of irritability is taken into consideration. Since trait irritability is dimensional, early emerging, transdiagnostic, and cuts across multiple domains of functioning (i.e., cognition, emotionality, behavior), this phenotype is well positioned to inform the RDoC matrix.

The neural and physiological mechanisms that contribute to irritability in early childhood have been described in detail else-where (Beauchaine et al., 2017; Brotman, Kircanski, & Leibenluft, 2017). In brief, high levels of irritability have been associated with psychobiological disruptions in Cognition×Emotion interactions, namely deficits in attention, threat detection, and reward processing, driven by aberrant activity in a range of frontal and subcortical regions (Brotman et al., 2017; Leibenluft, 2017; Wakschlag et al., 2018). Specific links between irritability and disrupted attentional processing, error monitoring, and inhibitory control have been observed. For example, high levels of irritability in infancy have been associated with poor behavioral inhibitory control, but only among infants with left frontal asymmetry, a putative indicator of approach-motivated tendencies measured via electroencephalography (EEG) (He et al., 2010). In addition, children who are highly irritable tend to show deficits in rapid attention capture (N1), conflict monitoring (N2), and allocation of attentional resources (P3), assessed via event-related potentials (ERPs), relative to their peers (Deveney et al., 2019; Rich et al., 2007). These children also show abnormal engagement of the anterior cingulate cortex (Perlman et al., 2015; Rich et al., 2011) and medial frontal gyrus (Adleman et al., 2011), areas critical for performance monitoring and attentional control. Collectively, results to date point to a pattern of cognitive dysfunction that may abet frustration and thwart effective self-regulation among children with high levels of irritability.

This pattern of cognitive dysfunction may also have an impact on emotional processing for these young children. Indeed, children high in irritability show biased attention toward and away from threat, impaired emotion labeling, and a lower threshold for interpreting a facial expression as angry (Brotman et al., 2017; Hommer et al., 2014; Salum et al., 2017; Stoddard et al., 2016), all similar to affect-biased attention patterns implicated in anxiety (e.g., Bar-Haim, Lamy, Pergamin, Bakermans-Kranenburg, & Van Ijzendoorn, 2007). Neuroimaging studies indicate that greater irritability is associated with aberrant amygdala activation during emotional face processing, although some studies show hypoactivation (Brotman et al., 2010) while others show hyperactivation (Thomas et al., 2013). These inconsistent findings may reflect non-linear brain–behavior relationships (Northoff & Tumati, 2019). For example, Grabell and colleagues (2018) found that lateral prefrontal cortex activation during frustration was positively correlated with irritability within the typical range, but negatively correlated for those with clinical levels of irritability. This finding highlights the necessity to examine irritability dimensionally to clarify unique neural mechanisms associated with lower or higher severity. A child’s age and developmental status may also play a role in this brain–behavior relationship. Tseng and colleagues (2019), for example, showed that after being frustrated, children high in irritability exhibit abnormal frontal and striatal engagement during a subsequent nonfrustrating attention-orienting task. This pattern of neural activation suggests that children prone to irritability may have difficulty shifting processing responses with shifts in context or events. Thus, prior frustrating events may spill over into subsequent behavior. Importantly, this association was stronger for children compared to adolescents, indicating that attentional impairment specific to emotional contexts may vary with age, and potentially with an increasing ability to regulate and engage in flexible behavior.

Highly irritable children also show disrupted reward learning, with difficulty detecting contingencies between responses and rewards, as well as impaired behavioral adaptation when contingencies change (e.g., Adleman et al., 2011; Dickstein et al., 2010). This effect is thought to be attributable to dopaminergic dysfunction in mesolimbic circuitry of the brain (Schultz, 2002; Tobler, Fiorillo, & Schultz, 2005), such that mismatches between reward predictions and outcomes are not adequately encoded. Evidence suggests that children high in irritability show elevated sensitivity to rewards, indexed by an exaggerated reward positivity, an ERP associated with medial prefrontal cortex and ventral striatum activation (Kessel et al., 2016). These children also show greater frustration accompanied by ventral striatum deactivation when an expected reward is denied (e.g., Deveney et al., 2013). Similarly, another study of school-aged children showed heightened anterior cingulate cortex activation when a reward was anticipated, and greater posterior cingulate engagement, a region involved in shifting the focus of attention from internal to external, when progress toward a reward was blocked (Perlman et al., 2015). These patterns suggest that highly irritable children may experience anticipated reward as more salient, and barriers toward reward as particularly aversive and/or unexpected.

Importantly, irritability during the first few years of life predicts greater reward sensitivity and proneness to frustration (e.g., Kessel et al., 2016) as well as reduced inhibitory control (He et al., 2010) later in childhood. Abnormalities in connectivity and reward-related activation of the amygdala and frontal regions, circuitry involved in emotion regulation and cognitive control, have been shown to differ based on the developmental timing of the emergence of high irritability (Dougherty et al., 2018). That is, irritability-linked frontal–amygdala connections were found to be more lateralized among highly irritable preschoolers, and more bilaterally distributed among irritable school-aged children. This suggests that early abberant neural activation may become more entrenched as regulatory circuitry matures, highlighting the importance of identifying high-risk phenotypes that emerge early in life.

Together, this pattern of neural, cognitive, and affective activation that associates with trait irritability in early childhood may contribute to increased reward (and novelty) motivation, as well as dysregulated, reactive, and impulsive behavior (Beauchaine et al., 2017; Wakschlag et al., 2018). It is important, however, to consider the environment in which this activation may occur. Sensitive caregiving, for example, has been shown to protect against increases in anger and aggressive behavior over time, whereas a mother’s history of antisocial behavior had the opposite effect (Perra et al., 2020; see also Hay, Pawlby, Waters, Perra, & Sharp, 2010, 2011, 2014; NICHD Early Child Care Research Network, 2004; Reuben et al., 2016; Robinson et al., 2011). Bidirectional associations between child irritability and negative parenting behaviors (i.e., non-responsiveness and inconsistent discipline) have also been identified (e.g., Lengua, 2006; van den Boom, 1989). Further, infant irritable distress has been shown to predict greater behavioral problems in toddlerhood, but only among those whose parents guided their attention toward the source of frustration (Crockenberg, Leerkes, & Bárrig Jó, 2008). Thus, irritable children may benefit from parents able to orient their child’s attention away from aversive stimuli, setting the stage for adaptive self-regulation. Critically, broad social contextual factors must also be considered when examining the link between irritability and caregiving behaviors. Socioeconomic status moderates the association between parental support and child negative emotionality such that stronger effects are detected among families with low as opposed to high socioeconomic status (Paulussen-Hoogeboom, Stams, Hermanns, & Peetsma, 2007). Irritability is also correlated with parental anxiety and depression (Dougherty et al., 2013a, 2013b), and adversity in early life, including maltreatment and poverty (Pagliaccio, Pine, Barch, Luby, & Leibenluft, 2018).

### Behavioral inhibition: A trait antecedent of acute threat

**Figure 2.**
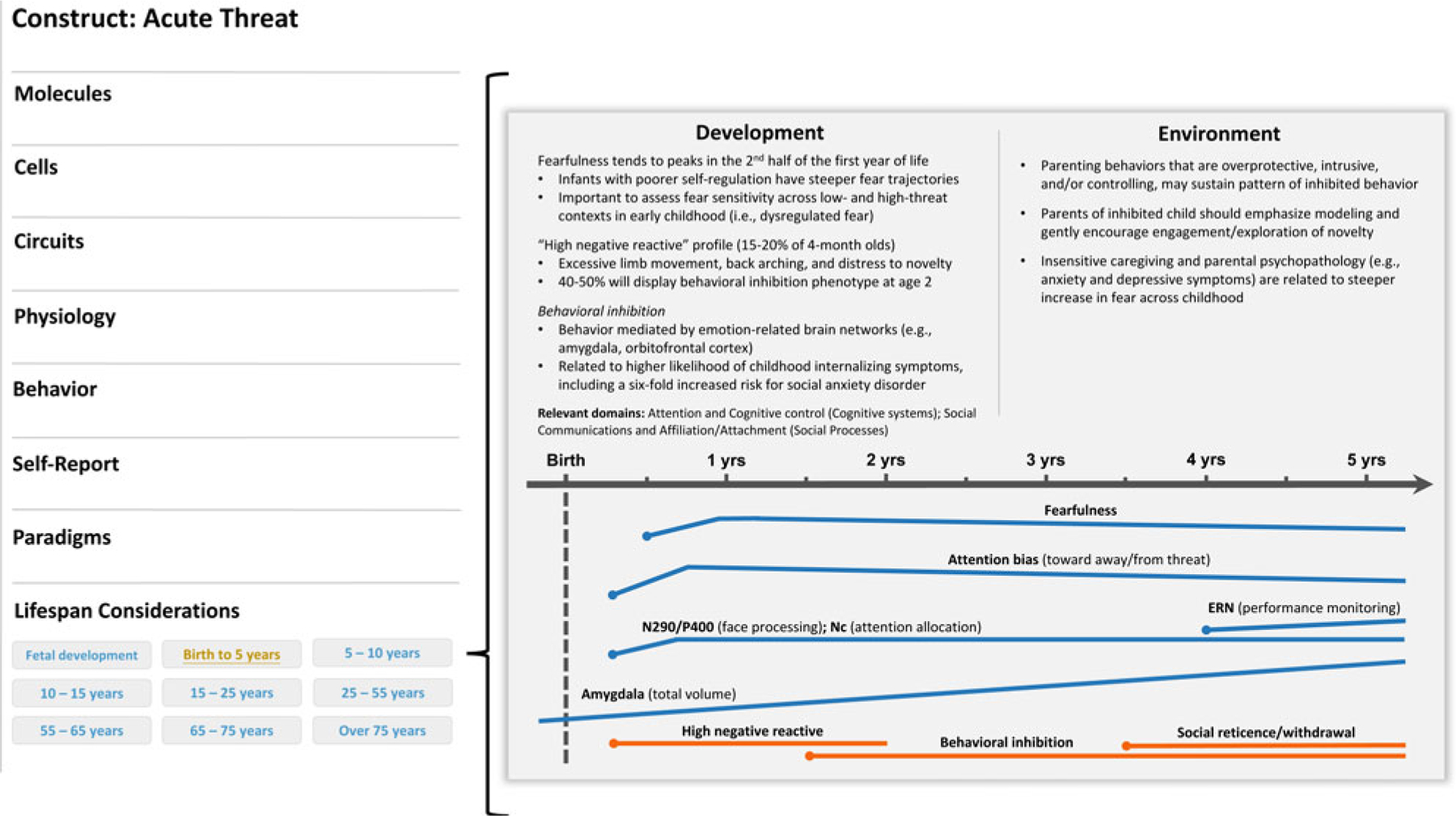
Illustrative example of a developmental qualifier for the acute threat (negative valence system) construct. A development subsection (“life span considerations”) could be added below the currently listed levels of analysis on the construct page (left panel). In this example, a researcher clicked on the “birth to 5 years” tab and was directed to a new screen that describes developmental considerations available for this construct in this age range. In this case, aspects of behavioral inhibition would be described and, when applicable, visually represented across time. The point in development when a construct could be reliably measured would be indicated on the timeline with a dot. Moreover, various colors could be incorporated to indicate normative and high-risk profiles or trajectories. Relevant cross-domain considerations would also be noted.

Acute threat (“fear”) is defined as a response to perceived danger that motivates defensive actions across systems to protect oneself (NIMH, 2018). Underpinned by overlapping neural mechanisms, behavioral inhibition is a possible antecedent to the acute threat construct evident in early childhood (Figure 2). Aspects of behavioral inhibition may also inform development of other RDoC constructs, chiefly the potential threat (“anxiety”) construct (negative valence systems). As noted above, some RDoC constructs lack specificity in their description of dysfunction, and at times overlap conceptually as well as across levels of analysis. For simplicity, we will only consider the link between behavioral inhibition and the acute threat construct. Nevertheless, the putative link between behavioral inhibition and the potential threat construct underscores the transdiagnostic utility of early emerging temperament traits, as well as the fact that matching temperament traits to RDoC constructs in a one-to-one manner is likely to be an over-simplification of the trait-based approach to psychopathology risk.

Infants who are more likely to be behaviorally inhibited in toddlerhood tend to display high levels of limb movement and distress in response to low-threat yet novel stimuli (e.g., a moving mobile), a phenotype referred to as “high negative reactive” (Fox et al., 2001; Garcia-Coll et al., 1984). Just under half of high-negative-reactive 4-month-old infants will go on to display the behavioral inhibition phenotype (Kagan, 2018). As these children age, the stimuli that evoke a distress response tend to move from undifferentiated novelty to social interactions or social cues, although the putative mechanisms underlying behavioral inhibition are thought to be constant over time. Social reticence, anxiety, and depression are common outcomes for children who were behaviorally inhibited as toddlers (Clauss & Blackford, 2012; Klein & Mumper, 2018). Most worthy of note, meta-analytic data indicate that over 40% of behaviorally inhibited young children had a social anxiety disorder as adolescents (Clauss & Blackford, 2012), lending support to the clinical utility of this early emerging phenotype.

Heterogeneity in this behavioral phenotype may be parsed further to improve the prediction of high-risk trajectories. To this end, Buss and colleagues have demonstrated the utility of considering context inappropriate fear reactivity as an indicator of elevated psychopathology risk above and beyond inhibited behavior (Buss, 2011; Buss et al., 2018). A core assumption of this approach is that adaptive (or maladaptive) behavior is a function of a young child’s appraisal of context-dependent threat, whether it be real or perceived. Assessment of fear reactivity across situations that varied from low- to high-threat is crucial, as toddlers who display dysregulated fear in low-threat situations cannot be distinguished from their inhibited peers in high-threat contexts.

Few temperament traits have been as extensively examined across levels of analysis as behavioral inhibition (Buss & Qu, 2018; Clauss et al., 2015). Behaviorally inhibited individuals show structural neural differences compared to their peers, including larger amygdala, caudate, and orbitofrontal cortex volume (Clauss et al., 2014; Hill, Tessner, Wang, Carter, & McDermott, 2010), increased thickness in the ventromedial prefrontal cortex (Schwartz et al., 2010), and decreased thickness in the dorsal anterior cingulate and lateral orbitofrontal cortices (Schwartz et al., 2010; Sylvester et al., 2016). In addition, burgeoning research into intrinsic functional connectivity indicates that behaviorally inhibited adults (Blackford et al., 2014; Roy et al., 2014) and children (Taber-Thomas, Morales, Hillary, & Pérez-Edgar, 2016) have increased connectivity in the salience network (e.g., dorsal anterior cingulate) and decreased amygdala connectivity with the anterior cingulate. Together, this pattern of activation points to dysfunction in both “bottom-up” (e.g., vigilance to potential threat) and “top-down” (e.g., regulation of amygdala reactivity) processing (Blackford, Clauss, & Benningfield, 2018). The pattern of activation differs when contextual or temporal factors are considered; we refer readers to Jarcho and Guyer (2018), who provide an illustrative example demonstrating how neurocognitive processing (e.g., salience network, mentalizing network) differ based on the environmental demands of a social interaction for a behaviorally inhibited child (e.g., decision to attend a party, engaging with unfamiliar peers).

Young children who are behaviorally inhibited exhibit distinct patterns of evoked neural activity associated with attentional control and conflict monitoring (e.g., Brooker & Buss, 2014; Lamm et al., 2014; McDermott et al., 2009; Thai, Taber-Thomas, & Pérez-Edgar, 2016), operationalized by two ERPs, the N2 and the error-related negativity. Physiologically, behaviorally inhibited young children show a pattern of right-biased activation of the frontal lobe (e.g., Calkins, Fox, & Marshall, 1996; Fox et al., 2001), larger pupil dilation (Kagan et al., 1987), higher skin-conductance levels (Gilissen, Koolstra, van Ijzendoorn, Bakermans-Kranenburg, & van der Veer, 2007; Scarpa, Raine, Venables, & Mednick, 1997), higher baseline cortisol levels (Essex, Klein, Slattery, Goldsmith, & Kalin, 2010; Pérez-Edgar, Schmidt, Henderson, Schulkin, & Fox, 2008; Smider et al., 2007), and greater reductions in parasympathetic-mediated cardiac physiology (Brooker et al., 2013; Buss et al., 2018). Physiological results have been mixed, however, particularly with regard to parasympathetic activity (see Buss & Qu, 2018 for detailed discussion).

Although a reliable predictor of psychopathology, it is important to remember that most behaviorally inhibited toddlers do not show clinically salient behavior problems in childhood, suggesting that internal and/or external factors may moderate trait expression over time. Attention is one particularly potent internal factor that affects the emergence and maintenance of behavioral inhibition, underscoring the importance of cross-domain considerations for trait development (Bell & Wolfe, 2004; Degnan & Fox, 2007). Affect-biased attention is a putative domain-general mechanism that describes the automatic process through which a child attends to motivationally salient stimuli. Attention to motivating aspects of the environment may be particularly relevant for maintaining psychopathology risk among behaviorally inhibited young children as it shapes the child’s subjective sense of the environment. We refer the reader to Morales, Fu, and Pérez-Edgar (2016) for a detailed discussion of affect-biased attention and socioemotional development. In brief, emerging evidence suggests that affect-based attention may moderate the link between behavioral inhibition and later socioemotional functioning, with stable patterns of attentional bias toward or away from salient stimuli (i.e., not reflecting change in context) serving to sustain a young child’s behavioral tendencies over time.

Although research has typically focused on attentional performance during a single task (e.g., Bar-Haim et al., 2007; Pérez-Edgar et al., 2011), emerging evidence suggests that stability of attention across multiple tasks is related to temperament traits, such as fearfulness, in early childhood (Fu & Pérez-Edgar, 2019). With this in mind, Vallorani and colleagues (2021) examined affect-based attention across multiple tasks in infancy (4- to 24-month-olds). Using a variable-centered method (factor analysis), the authors identified two factors that described affect-based attention: (a) an “engagement” factor characterized by increased dwell time to affective faces in the dot-probe and overlap tasks, along with decreased latency to affective faces in the vigilance task, and (b) a “disengagement” factor characterized by increased latency to probe and dwell time in the dot-probe and overlap tasks, respectively. Higher levels of maternal anxiety were related to less engagement with faces among infants. Using a person-centered method (latent profile analysis) these authors identified two homogeneous groups of infants: (a) a “vigilant” group characterized by more engagement with faces and a better ability to disengage, and (b) an “avoidant” group characterized by less engagement with faces and a poorer ability to disengage. Further, infants of mothers who reported more anxiety were more likely to be in the “vigilant” group if they also exhibited high levels of negative affect.

It is worth noting that the developmental mechanism through which the interplay between attention bias and negative affect in infancy influences the emergence of behavioral inhibition is an active area of debate. Field and Lester (2010) outlined three hypothetical models aimed at describing this attention–affect association, namely, the integral bias model, the moderation model, and the acquisition model. On the one hand, the integral bias model suggests that specific characteristics of the individual (e.g., temperament) determine the presentation and maintanence of attentional biases, not development. The moderation model, on the other, suggests that attentional biases are maintained over time, but only for individuals with specific characteristics, such as behavioral inhibition. For individuals without these characteristics, it is predicted that attentional biases will decrease over time. Lastly, the acquisition model suggests that specific developmental experiences will cause normative attentional biases to increase over time. Our research team is currently collecting data to test these proposed models (Burris et al., 2019; in press).

In terms of the caregiving context, infants of mothers who are less sensitive in a dyadic interaction, or who report more depressive symptoms, show a steeper increase in fear over early childhood (Braungart-Rieker et al., 2010; Gartstein et al., 2010). Sensitive caregiving for an inhibited child involves a greater emphasis on modeling and gently encouraging engagement with novelty, compared to their less inhibited peers (Hane, Cheah, Rubin, & Fox, 2008; Kiel, Premo, & Buss, 2016). It has been found that inhibited toddlers with intrusive, controlling, or over-protective mothers (e.g., excessive limits on independence and exploration) exhibit higher levels of social reticence at preschool age (Degnan, Henderson, Fox, & Rubin, 2008; Kiel et al., 2016; Mount, Crockenberg, Jó, & Wagar, 2010; Rubin, Burgess, & Hastings, 2002; Rubin, Cheah, & Fox, 2001; Rubin, Hastings, Stewart, Henderson, & Chen, 1997).

## Future Directions

We emphasized trait expression from birth to preschool age given that multiple overlapping neural, cognitive, emotional, motivational, and behavioral systems are being consolidated as a function of varied maturational and environmental influences. With this in mind, we offer three necessary next steps to infuse development (and temperament) into RDoC.

One future direction for RDoC involves the inclusion of measurement tools and guidelines that are appropriate for use with infants and young children. The RDoC matrix only lists a few paradigms suitable for children under the age of 5, such as the laboratory temperament assessment battery (LabTAB) (Goldsmith & Rothbart, 1996), the still-face paradigm (Tronick, Als, Adamson, Wise, & Brazelton, 1978), and the strange situation (Ainsworth, Blehar, Waters, & Wall, 1978). Developmental scientists, however, utilize a rich repertoire of behavioral tasks to understand early-life cognitive and emotional functioning. For example, LoBue and colleagues (LoBue & Deloache, 2008) provide support for a touch-screen detection paradigm that assesses attentional biases in young children. This method could be used with older individuals and/or in conjunction with methods from other units of analysis (e.g., electroencephalogram), thus exemplifying how a single paradigm can be utilized to measure a dynamic construct related to psychopathology risk from early childhood onward.

Measures that are valid, reliable, and can describe functioning across the life span will advance developmental research in the RDoC era. This is a challenging task given that trait expression often changes over time. Nevertheless, methodological approaches that identify risk traits or phenotypes and adapt mixed-method measurement instruments for various age groups across the life span will provide greater insight into how RDoC constructs develop. The Patient-Reported Outcome Measurement Information System, for example, identified risk traits (e.g., anger/irritability) and then adapted the measure in a developmentally sensitive manner for adults, children (5–17 years), and young children (1–5 years). As a result, researchers and clinicians may now administer a developmentally sensitive instrument to evaluate risk for psychological (and physical) health and dysfunction across the life span (Blackwell et al., 2020).

In relation to this challenge, the National Institutes of Health Toolbox is an existing database that outlines developmentally appropriate paradigms, and could seamlessly be integrated into the RDoC matrix with the addition of a “life span considerations” subsection. Temperament researchers and developmental psychopathologists have also sought to establish measures of temperament traits and clinical symptoms that can be considered from birth through adulthood (e.g., Achenbach, 2009; see Fu & Pérez-Edgar, 2015 for discussion of methodological approaches in temperament research). Paired with biological and behavioral (e.g., LabTAB) measures, these questionnaires may also prove useful in assessing psychopathology risk across the life span. If development is to be infused into RDoC, developmentally sensitive and psychometrically sound measures and procedures ought to be described in the RDoC matrix, possibly in the proposed “life span considerations” subsection.

Another future direction for both RDoC and early childhood temperament research may be to investigate the mechanisms underlying prenatal contributions to individual differences in cognitive and behavioral dysfunction, a critical next step for understanding intergenerational transmission of psychopathology risk. Although the neural, physiological, and behavioral correlates of early temperament have been well documented, the prenatal origins of these neurobehavioral differences remain largely unknown. Explanations that invoke only genetic or postnatal socialization pathways may not sufficiently explain the origins of temperament, as they fail to appreciate the ontogenesis of biological systems relevant to trait expression, particularly during periods of heightened susceptibility to environmental influences. Burgeoning evidence supports the influence of pre- and perinatal experiences as under-appreciated, experience-dependent contributors to individual differences in the biological underpinnings of temperament (see Van den Bergh et al., 2017 for review). For example, a recent study from Qiu and colleagues (2015) found that 6-month-old infants whose mothers reported higher levels of depressive symptoms while pregnant showed greater functional connectivity between the left amygdala and brain networks related to perception and regulation of emotions (e.g., anterior cingulate, insula, orbitofrontal cortex, temporal cortex). Elucidating the pre- and perinatal contributions (and associated mechanisms) to temperament in early childhood may inform RDoC on putative pathways underlying the intergenerational transmission of psychopathology risk.

Lastly, future research should consider how information on individual differences in early childhood temperament can bridge the gap with pediatric clinical practice in the RDoC era. Fortunately, clinical neurodevelopmental models that utilize information on temperamental tendencies already exist. Wakschlag and colleagues (2018), for example, outline a developmental specification framework, in which a child’s psychopathology risk is characterized as a function of neurodevelopmental predispositions (i.e., temperament traits) and skill acquisition (e.g., social perspective taking) that are evident in early childhood. This characterization provides researchers and clinicians with foundational information from which abnormal deviations in behavior may be determined and linked to dysfunction in cognitive and affective systems. This information may then be used to determine the clinical course, correlates, and prognosis (see Wakschlag et al., 2018 for example application with early childhood irritability and callousness). The inclusion of temperament in this neurodevelopmental model and, as we propose, in the RDoC matrix may provide a bridge between RDoC and front-line treatments for pediatric samples. This approach may ultimately guide clinicians toward malleable points of intervention to prevent the onset of psychopathology in later childhood via the identification of dysfunction in early cognitive and affective systems.

## Conclusions

RDoC is an innovative approach designed to explore dimensions of human behavior that move beyond the limits of psychiatric categories in the hope of aligning the identification of psychological health and dysfunction with clinical neuroscience. To advance this goal, we propose the incorporation of core principles of temperament theories into a new “life span considerations” subsection as one option for infusing development into the RDoC matrix. Temperament traits represent transdiagnostic vulnerabilities that transact with environmental influences to shape functional development for a young child. This proposal necessitates that researchers embrace complexity to understand the early life origins of psychopathology risk. In doing so, researchers and clinicians may ultimately have the tools necessary to support emotional development and reduce a young child’s likelihood of psychological dysfunction beginning in the first years of life.

## Supplementary Material

Supplement
