# Supplementary material for: The centrality of temperament to the research domain criteria (RDoC): The earliest building blocks of psychopathology
Source: Dev Psychopathol. Author manuscript; Available in PMC 2023 Mar 25. (PMC10039756; doi:10.1017/S0954579421000511)
Supplement: Supplement [file NIHMS1872651-supplement-Supplement.docx]

**Supplement Table 1.** Studies that used person-centered approaches to classify temperament phenotypes from 4-months through 5-years of age. Studies that examined the same sample at different time points are indicated by the “Longitudinal” note.

CBQ = Child Behavior Questionnaire (Rothbart, Ahadi, Hershey, & Fisher, 2001; EAS = EAS Temperament Survey (Buss & Plomin, 1984). IBQ = Infant Behavior Questionnaire (Rothbart, 1981). IBQ-R= Infant Behavior Questionnaire-Revised (Gartstein & Rothbart, 2003). LabTAB = Laboratory Temperament Assessment Battery (Goldsmith & Rothbart, 1996). RITQ = Revised Infant Temperament Questionnaire (Carey & McDevitt, 1978). TBAQ = Toddler Behavior Assessment Questionnaire (Goldsmith, 1996). QUIT = Questionari italiana del temperamento (Axia, 2002). NICHD SECCYD = National Institute of Child Health and Human Development Study of Early Child Care and Youth Development.

|  | **Age (months)** | ***N*** | **Sample** | **Measure** | **Profiles (% of sample)** |
| --- | --- | --- | --- | --- | --- |
| Lin et al. (2018) | 4 | 1085 | Prenatal substance exposure | IBQ | **4 profiles**  10% Reactive, well-regulated  13% Negative reactive, dysregulated  37% Mod. low reactive, mod. dysregulated  41% High positive affect, well-regulated |
| Komsi et al. (2006) | 6 | 231 | Community | IBQ | **3 profiles**  32% Resilient  34% Undercontrolled  34% Overcontrolled |
| Planalp & Goldsmith (2020) | 6  (Longitudinal) | 594 | Community | LabTAB | **4 profiles**  11% Positive/active  19% Withdrawn/inhibited  34% Typical  36% Low negative |
| Gartstein et al. (2017) | 3-8  (Longitudinal) | 731 | Community | IBQ-R | **3 profiles**  13% Frustrated/difficult to calm  39% Fearless/low positive  49% High positive/regulated |
| Sanson et al. (2009) | 4-8 | 2443 | Community | RITQ | **4 profiles**  20% Reactive/inhibited  25% Nonreactive/outgoing  26% High attention regulation  28% Poor attention regulation |
| Beekman et al. (2015) | 9  (Longitudinal) | 530 | Adoption | IBQ | **4 profiles**  13% Positive reactive  17% Negative reactive  28% Typical expressive  43% Typical low expressive |
| Gartstein et al. (2017) | 9-12  (Longitudinal) | 625 | Community | IBQ-R | **5 profiles**  13% Low positive  13% Low approach/difficult to calm  17% High positive/regulated  28% Avg. approach/avg. vocal reactivity  30% High active |
| Planalp & Goldsmith (2020) | 12 (Longitudinal) | 915 | Community | LabTAB | **4 profiles**  15% Withdrawn/inhibited  20% Low negative  21% Positive/active  44% Typical |
| Lin et al. (2018) | 12 | 333 | Prenatal substance exposure | IBQ | **4 profiles**  11% Negative reactive, dysregulated  21% High positive affect, well-regulated  28% Reactive, well-regulated  41% Mod. low reactive, mod. dysregulated |
| Ostlund et al. (*under review*) [this issue] | 16 | 308 | Prenatal substance exposure | TBAQ; LabTAB | **4 profiles**  16% Reactive, dysregulated  17% Well-regulated  26% High reactive  39% Low reactive |
| Jansen & Mathiesen (2008) | 18; 30;  36-48 | — | Community | EAS | **5 profiles**  % Inhibited  % Undercontrolled  % Uneasy  % Unremarkable  % Confident |
| Beekman et al. (2015) | 18  (Longitudinal) | 513 | Adoption | TBAQ | **4 profiles**  8% Fearful  17% Active reactive  35% Negative reactive  40% Positive reactive |
| Putnam & Stifter (2005) | 24 | 126 | Community | Obs. | **4 profiles**  2% Extremely inhibited  24% Inhibited  36% Low/low  38% Exuberant |
| Beekman et al. (2015) | 27  (Longitudinal) | 490 | Adoption | TBAQ | **4 profiles**  9% Active reactive  13% Fearful  30% Negative reactive  48% Positive reactive |
| Usai et al. (2009) | 28 | 106 | Community | QUIT | **3 profiles**  22% Inhibited  30% Inattentive  48% Typical |
| Caspi & Silva (1995) | 36 | 1023 | Community | Obs. | **5 profiles**  8% Inhibited  10% Undercontrolled  15% Reserved  27% Confident  40% Well-adjusted |
| Van den Akker et al. (2010) | 36-48 | 384 | Community | TBAQ | **3 profiles**  10% Fearful  31% Expressive  58% Typical |
| Prokasky et al. (2017) | 48 | 96;187;757 | Community/  NICHD SECCYD | CBQ | **6 profiles**  % Unregulated  % Regulated  % High reactive  % Bold  % Average  % Well-adjusted |
